# Association of dietary cholesterol and dyslipidemia in Chinese health examinees

**DOI:** 10.1186/s41043-022-00293-y

**Published:** 2022-05-03

**Authors:** Junqiang Pan, Wenqi Han, Yongrong Jiang, Jine Wu, Xin Zhou

**Affiliations:** 1 Department of Cardiovascular Medicine, Xi’an Central Hospital, 161 West Fifth Road, Xi’an, 710003 China; 2 National Engineering Research Center for Biomaterials, 29 Wangjiang Road, Chengdu, 610064 China; 3 Second Department of Cardiovascular Medicine, Shaanxi Provincial People’s Hospital, No.256 Youyi West Road, Xi’an, 710068 China; 4 Department of Cardiovascular Medicine, The First Affiliated Hospital of Xi’an Jiaotong University, 277 Yanta West Road, Xi’an, 710061 China

**Keywords:** Dietary cholesterol intake, Dyslipidemia, Chinese health examinees

## Abstract

**Background:**

The association between dietary cholesterol consumption and dyslipidemia is still in controversy. The study aims to evaluate whether dietary cholesterol intake associated with dyslipidemia and its components in Chinese health examinees.

**Methods:**

A large-scale cross-sectional study was conducted among health examinees of in Shaanxi province. Totally of 8358 participants (3677 male and 4681 female) were included. Dietary cholesterol intake was assessed by validated food frequency questionnaire. Multivariable regression and restricted cubic spline models were used to capture the linear and non-linear association between dietary cholesterol and dyslipidemia.

**Results:**

A total of 2429 (29.1%) subjects were newly diagnosed of dyslipidemia, the prevalence was 29.2% in male and 27.7% in female. Mean intake of dietary cholesterol was 213.7 mg/day. After adjusting for all potential confounders including demographics information and lifestyles, higher cholesterol consumption was related to lower risk of dyslipidemia, the ORs (95% CIs) across Q2 to Q4 group were 0.87 (0.60–1.26), 0.80 (0.55–1.18) and 0.61 (0.41–0.91) in female. With further controlling for nutrients principal components, a null association was observed between dietary cholesterol and dyslipidemia and serum lipids, regardless of gender. Results of restricted cubic splines showed that the risk of dyslipidemia decreased slowly until around 300 mg/day in men and 200 mg/day in women, although the non-linear association was not significant.

**Conclusions:**

The study suggested that dietary cholesterol consumption was not associated with dyslipidemia or serum lipids in Chinese health examinees, although a decreased risk was observed before the threshold points.

**Supplementary Information:**

The online version contains supplementary material available at 10.1186/s41043-022-00293-y.

## Background

Dyslipidemia is defined as the prevalence of adverse blood concentrations of any of the followings: triglycerides (TG), low-density lipoprotein cholesterol (LDL-C), total cholesterol (TC) and high-density lipoprotein cholesterol (HDL-C) [1]. It is one of leading contributors to cardiovascular disease and mortality in global [2]. It was reported that high concentration of TC contributed to approximately 4.4 million deaths and 93.8 million disability-adjusted life-years by Global Burden of Disease study [3]. Thus, the prevention of this disease was of great importance to public health.

In the past decades, limited dietary cholesterol consumption has been recommended initially for cardiovascular disease (CVD) prevention. Pooled study of large-scale prospective cohorts demonstrated that high cholesterol consumption was related to risk of mortality and CVD mortality or dyslipidemia [4, 5]. However, there has been a long-running debate on whether serum cholesterol levels are responsive to high intake of cholesterol [6]. Actually, reviewed studies are heterogeneous and lack the methodologic rigor to draw any conclusions regarding the effects of dietary cholesterol on CVD risk [7]. Therefore, the Chinese and 2015–2020 US dietary guidelines did not carry forward the upper limit for dietary cholesterol [8]. Recently, such public concern has been intensified by numerous population studies across continents that have demonstrated the positive association between dietary cholesterol intake and adverse health outcomes [5, 9, 10]. They also suggest that clinicians and policy makers should continue to highlight the restriction of cholesterol intake in the dietary recommendations. Yet, these work pay little attention on potential multicollinearity among nutrients and the independent health effect of cholesterol has been not elucidated. For instance, a negative association was reported after saturated fatty acid intake [11]. Furthermore, whether the association vary across cholesterol consumption level, remains unclear.

Given the inconsistencies among studies, the study was conducted to assess gender-specific association between dietary cholesterol intake and dyslipidemia. We hypothesized that dietary cholesterol intake might have significant association with risk of dyslipidemia and its components among Chinese health examinees. In addition, we also attempt to examine the possible non-linear association and seek information on threshold of dietary cholesterol.

## Methods and materials

### Ethics statement

The current study complied with the Declaration of Helsinki and the protocol was approved by the Ethics Review Committee of Xi’an Central Hospital. Informed written consent was obtained from each participant prior to participation.

### Study setting

Xi’an is the provincial capital of Shaanxi Province in northwest China. Between March 2013 and December 2017, health examinees were recruited to assess cardiovascular disease and its potential risk factors in Xi’an. Adult participants were asked to volunteer through telephone call, on-site invitation or mailed letters in one health examination center.

A total of 10,780 individual were recruited. We further excluded those with nutrition-related disease, including diabetes, stroke or hypertension (*n* = 1908). We made these exclusion to minimize the prevalence-incidence bias and the effect of reverse causality led by potential confounders such as lifestyle factors [12]. We further excluded subjects with missing information of physical examination, left food frequency questionnaire total blank or missing main food intake and individuals with energy intake < 500 or > 5000 kcal/day (*n* = 514). Ultimately, 8358 participants including 3677 male and 4681 female remained in the final analysis.

### Assessment of cholesterol intake

Dietary information was collected by a 92-item semi-quantitative food frequency questionnaire (FFQ), with nine intake frequency categories, including “almost never” to “ ≥ 3 times/day”. Our FFQ is established and revised based on the validated Xi'an FFQ in China [13]. In validation study, the deattenuated correlation coefficients for nutrients estimated by the FFQ and 3-d 24 dietary recalls were from 0.35 to 0.85 in men, and slightly lower in men [13]. Meanwhile, the correlation coefficients between the two FFQs in reproducibility study ranged from 0.41 to 0.68 in men and 0.36 to 0.66 in women.

During the FFQ investigation, participants were e-mailed or asked to recall the average portion and frequency of each food consumed during the last 12 months. Main nutrients intake, including carbohydrate, protein, fat, sodium and cholesterol intake were calculated according to Chinese Food Composition Table. When we explored the association between cholesterol intake and dyslipidemia, nutrients intake was caloric-adjusted to 1980 kcal/day (the mean intake of participants) by residual method [14].

### Covariates assessment

A standard self-administered questionnaire was used to collect socioeconomics and demographics information (age, marital status, education, occupation, income, transportation and so on), history of disease, several lifestyles (smoking and physical activity) and diet. The trained public health professionals charged for explaining the definition of all items.

Evidence from the large-scale epidemiological studies indicated rational nutrients intake or better nutrition status might be associated with higher socioeconomic position, healthier lifestyle factors and there might be multi-collinearity cross different nutrients [15, 16]. As a consequence, adjusting for them in multivariate regression models would further decrease potential residual confounding.

It is reported that many nutrients are significantly correlated, such as protein, fat, and dietary cholesterol intake. To avoid multicollinearity of nutrients in regression analyses, we used principal component analysis based on all potential nutrients (saturated fatty acid, polyunsaturated fatty acid, monounsaturated fatty acids, carbohydrate, animal protein, plant protein, fiber, calcium and potassium). The first two principal components explained 73.6%. The first component exhibited the factor loadings of saturated fatty acid, polyunsaturated fatty acid, monounsaturated fatty acids, and animal protein more than 0.4. The correlation between the second component and other nutrients ranged from 0.32 to 0.90. And we took the two principal components as potential nutrition confounders in regression models.

### Definition of outcomes

The participants were asked to stay fast for ≥ 8 h before the medical examination. Serum lipids were analyzed by automatic biochemical analyzer. The diagnosis criteria was based on the According to the Chinese adult dyslipidemia prevention guide (2016 edition) [17], which defined dyslipidemia as one or more of the following 4 indicators, hypercholesterolemia (TC ≥ 6.20 mmol/L); hypertriglyc-eridemia (TG ≥ 2.30 mmol/L); low levels of HDL-C (HDL-C < 1.04 mmol/L); high levels of LDL-C (LDL-C ≥ 4.14 mmol/L).

### Statistical analysis

In current analysis, we created a cross product of sex and cholesterol intake to assess the possible interaction. This variable was significantly associated with dyslipidemia in regression model (Wald *χ*^2^ = 13.985, *P* < 0.001). Therefore, gender-specific analysis was conducted to better understand the relationship. Demographic and lifestyle information were summarized by percentage for categorical variables across quartiles of dietary cholesterol intake. Group difference was tested by analysis of variance or chi-square test. Correlation analysis was performed to describe the association between dietary cholesterol and main food and nutrients. Multivariable logistic regression models were used to estimate odds ratios (ORs) of dyslipidemia and their 95% confidence intervals (CIs), with the lowest intake group (the first quartile, Q1) as reference. Model 1 adjusted for energy, age, education and income level. Model 2 adjusted for the variables in model 1 plus physical activity level, alcohol intake, smoke status and BMI. Model 3 adjusted for the variables in model 2 plus two nutrients principal components. The linear trend across quartiles was assessed by using the median value of each quartile as a single continuous variable and entering into the regression models [18]. Restricted cubic spline (RCS) with three knots (25th, 50th, 75th percentiles) was modeled to explore the potential non-linear association, with the median value as the reference. Multivariable linear regression was conducted to evaluate the influence of cholesterol intake by increment of one S.D. (50 mg/day) on serum lipids. In exploratory analysis, we also examined the association between main food source and dyslipidemia in regression models. We also repeated regression models when analytical sample merely includes those participants who join in the survey the first time. Two-sided *P* < 0.05 was considered as statistically significant. The RCS analysis was performed in STATA 12.0, other were performed with SPSS version 18.0 (SPSS Inc, Chicago, USA).

## Results

A total of 2429 (29.1%) subjects were diagnosed of dyslipidemia, the prevalence was 29.2% in male and 27.7% in female. The mean intake of dietary cholesterol was 213.7 mg/day, men consumed much more than women (243.5 vs. 197.3). Totally 24.1% of participants consumed more than 300 mg/day, the proportions were 31.0% in male and 20.5% in female. And 31.4% (27.1% of male and 33.6% of female) consumed cholesterol less than 100 mg/day. Additionally, cholesterol intake was positively associated with total fat, saturated fatty acid, monounsaturated fatty acids, polyunsaturated fatty acids, total protein and animal protein, but negatively associated with carbohydrate and fiber intake (Additional file 1: Table S1). Egg consumption contributed to 64.75% of the total cholesterol consumption while intake of red meat and other foods contributed 11.98% and 15.88%, respectively.

The main characteristic of the participants were presented according to quartiles of cholesterol consumption in Table 1. High cholesterol consumers tend to be younger, smokers, in lower BMI, and better educated and consume more energy and fat.

**Table 1:** Baseline characteristics according to cholesterol consumption in men and women

	Q1	Q2	Q3	Q4	P*
Cholesterol (mg/day)	< 90.0	90.0–144.6	144.6–258.8	> 258.8	
No. of subjects	2090	2089	2090	2089	
Male (%)	31.0	36.9	42.7	55.2	< 0.001
Age (year)	49.7 ± 10.7	48.3 ± 11.5	47.1 ± 11.9	47.2 ± 12.0	< 0.001
BMI (kg/m^2^)	22.7 ± 3.0	22.7 ± 2.8	22.3 ± 2.7	22.3 ± 2.8	0.008
Schooling year > 9 years (%)	7.7	10.9	14.8	13.2	< 0.001
Current drinker (%)	14.3	16.0	17.7	17.5	0.168
Current smoker (%)	11.0	14.6	19.3	21.7	< 0.001
Physical activity level (%)					< 0.001
Light	10.3	16.4	16.7	21.2	
Moderate	19.7	20.4	20.3	19.0	
High	73.0	63.2	62.9	59.8	
Nutrient intake^a^					
Energy (kcal/day)	1790.2 ± 666.3	1893.5 ± 703.7	1943.8 ± 742.6	1894.2 ± 672.5	0.002
Fat (g/day)	52.0 ± 16.4	74.6 ± 21.8	76.7 ± 24.7	75.3 ± 23.3	< 0.001
Protein (g/day)	41.9 ± 9.2	38.7 ± 10.8	41.5 ± 10.4	47.9 ± 10.6	< 0.001
Carbohydrate (g/day)	251.2 ± 53.5	203.6 ± 58.4	199.4 ± 56.4	201.5 ± 56.2	< 0.001
Sodium (mg/day)	5415.4 ± 3286.2	5819.8 ± 4098.4	5498.1 ± 3530.4	5408.9 ± 3560.9	0.188

Values were mean ± SD. or %

*P value was assessed by chi-square for categorical variables or by ANOVA for continuous variables

^a^Adjusted for total calorie intake, except energy daily intake

Table 2 presents the associations between cholesterol intake and dyslipidemia by gender. In females, significantly negative association was observed between cholesterol intake and dyslipidemia in model 1. The corresponding ORs (95% CIs) of Q2-Q4 were 0.82 (0.55–1.21), 0.75 (0.50–1.12) and 0.65 (0.43–0.98). And the relationship persisted to the edge of significance after further adjustment for physical activity level, alcohol intake, smoke status and BMI (*P*-trend = 0.049). However, cholesterol intake was not associated with dyslipidemia in model 3 after further adjustment for two nutrients principal components (*P*-trend = 0.109). In males, no significant association was detected in males.

**Table 2:** Adjusted odd ratios (95%CI) of dyslipidemia by cholesterol daily intake

	Q1	Q2	Q3	Q4	*P*-trend
Male					
Dyslipidemia prevalence (%)	35.7	29.7	28.6	32.3	
Intake (mg/day)	< 104.0	104.0–167.7	167.7–313.3	> 313.4	
Model 1	1.00	0.86 (0.52–1.42)	0.56 (0.33–0.95)	0.75 (0.45–1.24)	0.181
Model 2	1.00	0.87 (0.52–1.43)	0.54 (0.31–0.92)	0.74 (0.45–1.23)	0.138
Model 3	1.00	0.88 (0.51–1.53)	0.54 (0.31–1.01)	0.79 (0.45–1.40)	0.441
Female					
Dyslipidemia prevalence (%)	34.0	28.7	24.9	20.1	
Intake (mg/day)	< 85.9	85.9–133.4	133.4–234.8	> 234.8	
Model 1	1.00	0.82 (0.55–1.21)	0.75 (0.50–1.12)	0.65 (0.43–0.98)	0.044
Model 2	1.00	0.87 (0.60–1.26)	0.80 (0.55–1.18)	0.61 (0.41–0.91)	0.049
Model3	1.00	0.95 (0.62–1.45)	0.85 (0.55–1.32)	0.69 (0.44–1.08)	0.109

Model adjustments: Model 1: adjusted for energy, age, education and income level

Model 2: model 1 and further adjusted for physical activity level, alcohol intake, smoke status and BMI. Model 3: model 2 and further adjusted for two nutrients principal components

**Fig. 1:**
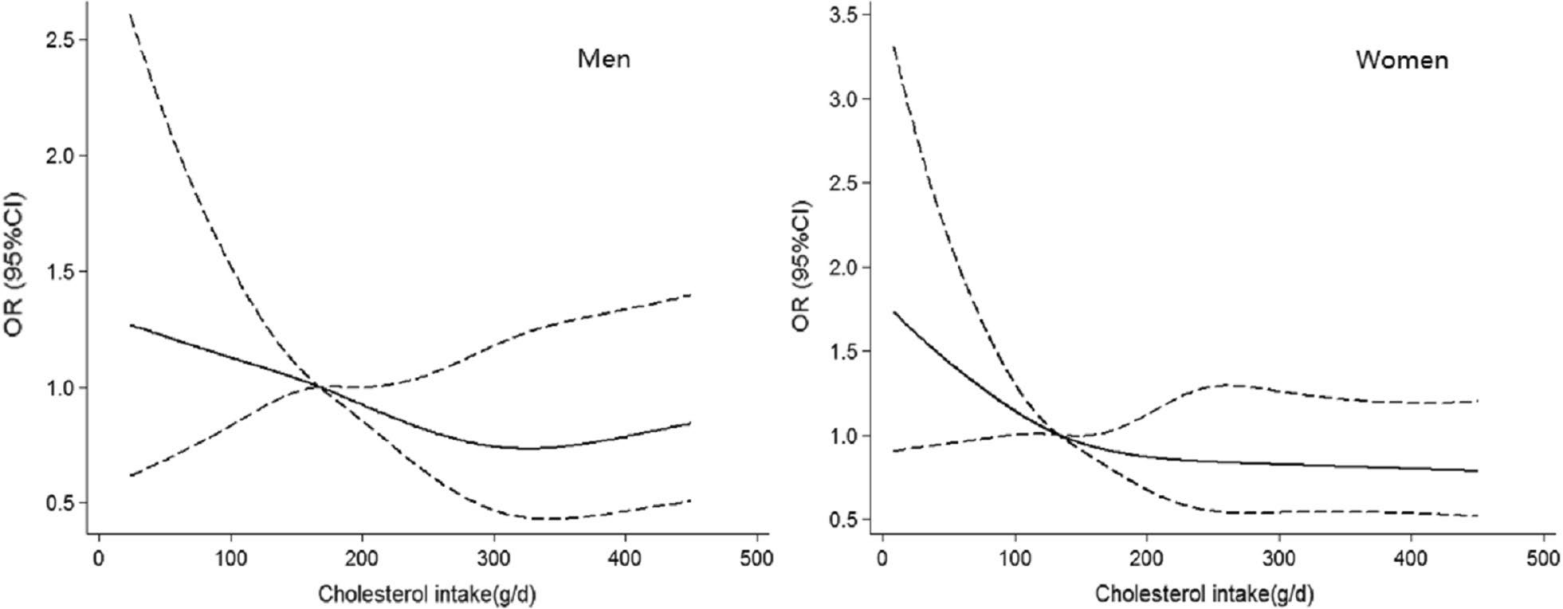
Non-linear dose–response association between dietary cholesterol consumption and dysilipidemia. Adjusted for energy, age, education, fortune index, BMI, physical activity level, alcohol intake and smoke status, two nutrient principal components. The figure in left was in men and the right in women

In Fig. 1 we used RCS analysis to model and visualize the relation of dietary cholesterol consumption and dyslipidemia. The median of cholesterol intake (167.7 g/day in men and 133.4 in female) was treated as reference. In men, the risk of dyslipidemia decreased slowly until around 300 mg/day and started to be flat afterward. In women, the point was brought forward to 200 mg/day. However, the non-linear associations did not reach statistical significance, irrespective of the gender (P > 0.05).

The linear regression was conducted to assess the association between cholesterol intake and parameters of serum lipid (Table 3). Cholesterol intake was associated with increased HDL (*β* ± SE: 0.010 ± 0.004, *P* = 0.020) and decreased TG (*β* ± SE: − 0.028 ± 0.012, *P* = 0.018) in women, after adjusting for same covariates above in model 2. However, the association decreased to be not significant when further adjusting for two nutrients principal components. No association was observed between cholesterol intake and serum lipids in men. In exploratory analysis, we estimated the relation between main food source of dietary cholesterol and dyslipidemia. We observed a contrary tendency between egg consumption and risk of dyslipidemia, although the association attenuated to be insignificance after controlling for lifestyle factors (*P* value = 0.058) (Additional file 1: Table S2). No relation was observed for other foods. Additionally, the number of participants entered the survey again ranged from 236 to 515 between 2013 and 2017. Similar results for dietary cholesterol intake and dyslipidemia were obtain when analytical sample merely includes those participants who join in the survey the first time (Data not shown).

**Table 3:** Association between cholesterol intake with fasting glucose and parameters of serum lipid by increment of 50 mg/day

	Model 1		Model 2		Model 3	
	*β* coefficient ± SE	*P* value	*β* coefficient ± SE	*P* value	*β* coefficient ± SE	*P* value
Male						
TC	− 0.007 ± 0.013	0.664	− 0.008 ± 0.013	0.546	− 0.005 ± 0.014	0.713
TG	− 0.021 ± 0.031	0.488	− 0.022 ± 0.031	0.474	− 0.018 ± 0.030	0.543
HDL	0.007 ± 0.005	0.204	0.007 ± 0.005	0.210	0.005 ± 0.005	0.372
LDL	− 0.013 ± 0.012	0.264	− 0.014 ± 0.012	0.221	− 0.011 ± 0.012	0.360
Female						
TC	− 0.014 ± 0.010	0.188	− 0.010 ± 0.011	0.361	− 0.013 ± 0.011	0.241
TG	− 0.030 ± 0.012	0.013	− 0.028 ± 0.012	0.018	− 0.018 ± 0.012	0.146
HDL	0.010 ± 0.004	0.019	0.010 ± 0.004	0.020	0.005 ± 0.004	0.198
LDL	− 0.016 ± 0.009	0.089	− 0.013 ± 0.009	0.150	− 0.012 ± 0.009	0.183

Model adjustments: Model 1: adjusted for energy, age, education and income level. Model 2: model 1 and further adjusted for physical activity level, alcohol intake, smoke status and BMI. Model 3: model 2 and further adjusted for two nutrients principal components

## Discussion

The current study demonstrated that cholesterol intake was negatively associated with prevalence of dyslipidemia and abnormalities in TG and HDL-C levels before adjusting for nutrient components, but not after adjusting for them in women. In addition, relatively high consumption was related to lower risk before 300 mg/day in men and 200 mg/day in women. Consumption of main food sources of cholesterol including egg, red meat might not have adverse effect on serum lipids. Yet, further large-scale prospective cohort studies are warranted to confirm the findings.

Consistent with previous studies [19, 20], the current analysis indicated a null association between cholesterol intake and risk of dyslipidemia or serum lipid levels in multivariable regression models with adjustment for nutrient`, irrespective of gender. In fact`, the negative association weekend into insignificance after controlling for nutrients in women. Similar results were reported in the Framingham offspring study and Korea national survey [11, 19] that no associations were detected after adjusting for fatty acids. Conversely, other two studies showed a positive association between dietary and LDL-C and TC, without adjusting for fatty acid [5, 9]. It was suggested that that fatty acids such as saturated fatty acid might stimulate or mediate the hepatic biosynthesis of cholesterol [19]. Our study supported the evidence for the high multicollinearity among nutrients, due to significant positive correlation between dietary cholesterol and three fatty acids and protein. And it was necessary to include main relative nutrients when assessing the independent health effect of dietary cholesterol. Also, the recent prospective cohort study among US populations found that additional 300 mg per day of cholesterol consumption was associated with higher risk of mortality, independent of diet quality and fat consumption [4]. This discrepancy was also indicative of the wide variability in cholesterol consumption patterns and suggested that cholesterol consumption at disparate eating dietary pattern might be associated with inconsistent health outcomes. In the aforementioned study, overall mean dietary cholesterol consumption was 285 mg per day, much higher than our study (213.7 mg per day). Furthermore, in US populations, meat consumption contributed 42% to the total dietary cholesterol and egg consumption contributed 25% [21]. While in Chinese adults, egg contributed more than half of dietary cholesterol (53.8%) and meat consumption contributed 26.0% [22]. In addition, cholesterol absorption efficiency from diet shows a large range from 15 to 85% between individuals [23], which might partly explained the mainly non-significant association of serum lipids.

We also tried to estimate the non-linear association between cholesterol consumption and dyslipidemia using RCS analysis. Dietary cholesterol intake was inversely associated with lower risk dyslipidemia before 200 mg/day in women and 300 mg/day in men, and with little association above the points. The result might provide a new direction to examine the health effect of dietary cholesterol, although the models did not achieve statistical significance. Meanwhile, the low proportion of participants consuming high cholesterol intake (300 mg/day) made it impossible to fully assess its dose–response relation with dyslipidemia, especially at high intake level. Thus, further study covering a wide range of cholesterol intake were essential to verify the finding.

No relation was found between main food sources of cholesterol and serum lipid levels. Previous research showed that the association between cholesterol and dyslipidemia might mainly attributed to egg consumption, the primary source of cholesterol [24]. Mechanistically, eggs were in rich in many other nutrients such as protein, choline and vitamins that might have implicated in dyslipidemia in different pathways [4]. In current study, consumption of red meat was not associated with abnormalities in serum lipids level, consistent with finding of meta-analysis concluding randomized controlled trials [25]. However, we could not explore the potential reasons limited by data.

In present study, all clinical measurements were achieved by train personnel through standardized procedures, which reduced measurement error. However, there were several limitation should be noted. First, the cross-sectional design led to inability to assess causality between cholesterol intake and health outcomes. Second, residual confounding might still exist, although many statistical analysis models were built, and those with history of chronic disease were excluded. Third, the generalizability of these findings might be limited as the study population was not general individuals but health examinees. Another major limitation was that we did consider the potential confounding of individuals who joined the study more than one time, however, these subjects accounted for little proportion and similar insignificant association were observed when they were excluded. Fifth, measurement error could not be avoided when the FFQ was used for dietary assessment, due to the inability to estimate exact portion sizes of food items. Whereas, the FFQ was still the most common and robust instrument for estimating long-term period diet information on individual level in large nutritional epidemiology studies, compared with other assessments [26]. Finally, the complex multicollinearity among nutrient would not be totally avoided, although we derived two nutrients principal components. Yet, large-scale prospective cohort studies covering a wide range of cholesterol intake would be required confirm the findings.

## Conclusions

In conclusion, this study indicates cholesterol intake was not associated with high prevalence of dyslipidemia and its partial components, regardless of gender. High dietary cholesterol consumption tend to decrease risk of dyslipidemia before 300 mg/day in men and 200 mg/day in women. There is no doubt that the dietary pattern and cholesterol level should be considered when interpreting the findings. Further long-term research is warranted among general population to conform the conclusion.

## Supplementary Information

**Additional file 1: Table S1.** The correlation coefficient between cholesterol intake and food and other nutrients. **Table S2.** The association between main food source of cholesterol and dyslipidemia.

## Data Availability

The datasets generated and/or analyzed during the current study are not publicly available but are available from the corresponding author on reasonable request.

## Abbreviations

BMI: Body mass index
CI: Confidence interval
CVD: Cardiovascular disease
FFQ: Frequency food questionnaire
HCL-C: High density lipoprotein cholesterol
LDL-C: Low density lipoprotein cholesterol
OR: Odd ratio
RCS: Restricted cubic spline
TC: Total cholesterol
TG: Triglyceride
