# Supplementary material for: Association of dietary cholesterol and dyslipidemia in Chinese health examinees
Source: J Health Popul Nutr. 2022 May 3;41:15. doi: 10.1186/s41043-022-00293-y (PMC9066786; doi:10.1186/s41043-022-00293-y)
Supplement: Supplementary file 1 — Additional file 1: Table S1. The correlation coefficient between cholesterol intake and food and other nutrients. Table S2. The association between main food source of cholesterol and dyslipidemia. [file 41043_2022_293_MOESM1_ESM.doc]

**Supplementary table 1. The correlation coefficient between cholesterol intake and food and other nutrients**

|  | Correlation coefficient | *P* value |
| --- | --- | --- |
| Food group |  |  |
| Egg | 0.872 | <0.001 |
| Red meat | 0.165 | <0.001 |
| Chicken | 0.104 | <0.001 |
| Dairy products | 0.166 | <0.001 |
| Nutrients |  |  |
| Fat | 0.215 | <0.001 |
| Saturated fatty acid | 0.343 | <0.001 |
| Monounsaturated fatty acids | 0.185 | <0.001 |
| Polyunsaturated fatty acids | 0.083 | <0.001 |
| Carbohydrate | -0.190 | <0.001 |
| Protein | 0.323 | <0.001 |
| Animal protein | 0.735 | <0.001 |
| Fiber | -0.083 | <0.001 |
| Sodium | -0.008 | 0.712 |

**Supplementary table 2. The association between main food source of cholesterol and dyslipidemia**

|  |  | <median | >median | *P* value |
| --- | --- | --- | --- | --- |
| All participants | Egg, g/d | <7.0 | >7.0 |  |
|  | Model 1 | 1.00 | 0.77(0.62-0.96) | 0.021 |
|  | Model 2 | 1.00 | 0.80(0.64-1.01) | 0.058 |
|  | Red meat, g/d | <23.33 | >23.33 |  |
|  | Model 1 | 1.00 | 0.88(0.70-1.12) | 0.302 |
|  | Model2 | 1.00 | 0.83(0.66-1.06) | 0.141 |
|  | Plant oil, g/d | <4.2 | >4.2 |  |
|  | Model 1 | 1.00 | 0.80(0.63-1.01) | 0.057 |
|  | Model2 | 1.00 | 0.87(0.68-1.10) | 0.253 |

§ Model adjustments: Model 1: adjusted for energy, age, education and income level.

Model 2: model 1 and further adjusted for physical activity level, alcohol intake, smoke status and BMI.
